# Effects of Antidepressants on IP-10 Production in LPS-Activated THP-1 Human Monocytes

**DOI:** 10.3390/ijms150813223

**Published:** 2014-07-28

**Authors:** Jui-Hsiu Tsai, Chang-Hung Kuo, Pinchen Yang, Kuang-Hung Cheng, Peng-Wei Wang, Cheng-Chung Chen, Chih-Hsing Hung

**Affiliations:** 1 Department of Psychiatry, Kaohsiung Municipal Ta-Tung Hospital, Kaohsiung Medical University Hospital, Kaohsiung Medical University, Kaohsiung 80761, Taiwan; E-Mail: d880340@kmu.edu.tw; 2 Department of Pediatrics, Kaohsiung Municipal Ta-Tung Hospital, Kaohsiung Medical University Hospital, Kaohsiung Medical University, Kaohsiung 80761, Taiwan; E-Mail: kuochanghung.dr@gmail.com; 3 Department of Psychiatry, Kaohsiung Medical University Hospital, Kaohsiung Medical University, Kaohsiung 80761, Taiwan; E-Mails: pinchen@kmu.edu.tw (P.Y.); 990107@mail.hmuh.org.tw (P.-W.W.); 4 Institute of Biomedical Science, National Sun Yat-Sen University, Kaohsiung 80424, Taiwan; E-Mail: khcheng@faculty.nsysu.edu.tw; 5 Department of Community Psychiatry, Kai-Suan Psychiatric Hospital, 130 Kai-Suan 2nd Road, Kaohsiung 80276, Taiwan; E-Mail: ccchen@kcg.gov.tw; 6 Department of Pediatrics, Kaohsiung Municipal Hsiao-Kang Hospital, Kaohsiung Medical University, Kaohsiung 81267, Taiwan

**Keywords:** antidepressants, IP-10, chemokine, monocyte, LPS

## Abstract

Major depressive disorder and cardiovascular disease are common serious illnesses worldwide. Selective serotonin reuptake inhibitors and norepinephrine-dopamine reuptake inhibitors may reduce the mortality of cardiovascular disease patients with comorbid depression. Interferon-γ-inducible protein 10 (IP-10), a type 1 T helper cell (Th1)-related chemokine, contributes to manifestations of atherosclerosis during cardiovascular inflammations; however, the pathophysiological mechanisms linking cardiovascular disease and effective antidepressants have remained elusive. We investigated the *in vitro* effects of six different classes of antidepressants on the IP-10 chemokine expression in lipopolysaccharide (LPS)-stimulated monocytes, and their detailed intracellular mechanisms. The human monocytes were pretreated with antidepressants (10^−8^–10^−5^ M) before LPS-stimulation. IP-10 was measured by enzyme-linked immunosorbent assay (ELISA) and then intracellular signaling was investigated using Western blotting and chromatin immunoprecipitation. Fluoxetine and bupropion suppressed LPS-induced IP-10 expression in monocytes, and they had no cytotoxic effects. Furthermore, fluoxetine inhibited LPS-induced IP-10 expression via the mitogen-activated protein kinase (MAPK)-p38 pathway. Fluoxetine and bupropion could not only treat depression but also reduce Th1-related chemokine IP-10 production in human monocytes. Our results may indicate a possible mechanism related to how particular antidepressants reduce the risk of cardiovascular disease.

## 1. Introduction

Major depressive disorder (MDD) has the highest lifetime prevalence (average 12%) of all psychiatric disorders [1]. In-patients with MDD have a lifetime prevalence of suicide of 8.6%, compared with less than 0.5% in the general population [2]. Cardiovascular disease (CVD) is also highly prevalent in adult populations, especially in the West, and is now the leading cause of death worldwide [3]. MDD and CVD frequently occur together, and result in increased cardiovascular mortality [4,5,6,7]. Given the detrimental effect of the two together, we need further understanding of the pathophysiological mechanisms that exist between MDD and CVD.

CVD is currently thought to be a chronic inflammatory disorder, and MDD has been proposed to be an inflammatory condition now that at least three meta-analyses [8,9,10] have shown that groups of individuals with MDD demonstrated increased levels of a variety of peripheral inflammatory biomarkers, compared with groups of non-depressed controls. However, inflammation is neither necessary nor sufficient to cause MDD. Individuals with increased inflammation represent just a biologically pertinent subtype of depression in which immune processes are especially relevant to disease development. It is better to conceive of inflammatory pathways as fully integrated into larger mind–body systems that have evolved to cope with environmental danger; hence, low levels of inflammatory stimulation can become depressogenic in individuals with vulnerability patterns in nonimmune elements of the larger systems [11].

Cytokines, including interleukin-1 (IL-1), tumor necrosis factor-á (TNF-á) and interleukin-6 (IL-6), which mediate the innate immune response, appear to be some of the most reliable peripheral biomarkers in MDD [10]. Patients treated with interferon-á (IFN-á) as a source of chronic cytokine exposure can produce a depressive syndrome with similarity to idiopathic MDD [12,13]. IFN-á-induced increases in IL-6 and TNF-á signaling have been shown to correlate with increases in depressive symptoms [14,15]. It has also been observed that short-term immune stimuli, e.g., low-dose lipopolysaccharides (LPS), are able to produce the emotional symptoms of depression/anxiety, even while being insufficient to induce the full-blown symptoms of sickness [16,17].

Given that increased production of proinflammatory cytokines is hypothesized to play a role in the etiology of MDD, treatment with antidepressants has long been proposed to have a negative immunoregulatory effect. An accumulating body of data [8,18,19] supports the proposition that antidepressants have effects on the production of proinflammatory cytokines, e.g., interferon-γ (IFN-γ), and negative immunoregulatory cytokines and agents, e.g., interleukin-10 (IL-10). It has been shown that the various types of antidepressant drugs, including tricyclic antidepressants (TCA), selective serotonin reuptake inhibitors (SSRI), heterocyclic antidepressants (HCA), serotonin/noradrenaline reuptake inhibitors (SNRI), and reversible inhibitors of monoamine oxidase A (RIMA), significantly suppress the ratio of IFN-γ/IL-10 and increase the production of IL-10 by peripheral blood leukocytes [19].

While most of the previous research has paid a great deal of attention to cytokine expression as a key mediator of the inflammatory process during the administration of antidepressants, little has been explored in the domain of chemokines. Chemokines are small glycoproteins that cause chemoattraction and activation of leukocytes to sites of immune reactivity [20]. In the innate immune system, monocytes are a major contributor to chemokine and cytokine production in response to LPS, which mainly consists of the outer membrane of Gram-negative bacteria. The intracellular mechanisms of LPS-induced inflammation have complex signaling pathways, of which, the most ubiquitous and best understood ones are the IκB kinase (IKK)-NF-κB pathway and three mitogen-activated protein kinase (MAPK) pathways [21]. The interferon-γ-inducible protein 10 (IP-10) is a T helper cell type 1 (Th1)-related chemokine that was identified as an early response gene induced by INF-γ in U937 monocytes [22]. Increased expression of IP-10 in patients with coronary disease has been directly associated clinically with restenosis after percutaneous coronary interventions [23]. IP-10 is also a potent endogenous inhibitor of angiogenesis and proved to be related to manifestations of atherosclerosis in the cardiovascular inflammatory process [22,24].

We hypothesized that the antidepressants with protective effects against CVD may modulate the effects of Th1-related chemokine IP-10 expression in human monocytes. In the present study, we investigated the *in vitro* effects of six different classes of antidepressants on the IP-10 chemokine expression in LPS-stimulated monocytes, and also explored the detailed intracellular mechanism.

## 2. Results and Discussion

### 2.1. Results

#### 2.1.1. S-Fluoxetine Suppressed Lipopolysaccharide (LPS)-Induced Interferon-γ-Inducible Protein 10 (IP-10) Expression in THP-1 Cells

**Figure 1:**
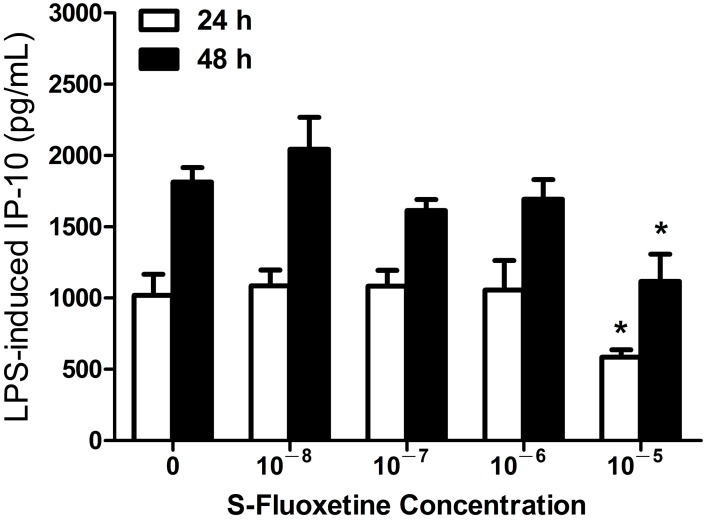
S-fluoxetine (10^−5^ M) suppressed lipopolysaccharide (LPS)-induced Interferon-γ-inducible protein 10 (IP-10) expression in THP-1 cells at 24 and 48 h after LPS (0.2 µg/mL) stimulation. * *p* < 0.05 compared with the control (LPS-untreated cells).

To examine the potential effect of S-fluoxetine on the expression of IP-10 in human monocytes, THP-1 cells were pretreated with varying doses of S-fluoxetine for 2 h and then stimulated with LPS. LPS-induced IP-10 production in THP-1 cells was significantly suppressed in the presence of S-fluoxetine (10^−5^ M after 24 and 48 h of LPS stimulation, both *p* < 0.05) (Figure 1). S-fluoxetine alone had no effect on IP-10 production (data not shown).

#### 2.1.2. R-Fluoxetine also Suppressed LPS-Induced IP-10 Expression in THP-1 Cells

**Figure 2:**
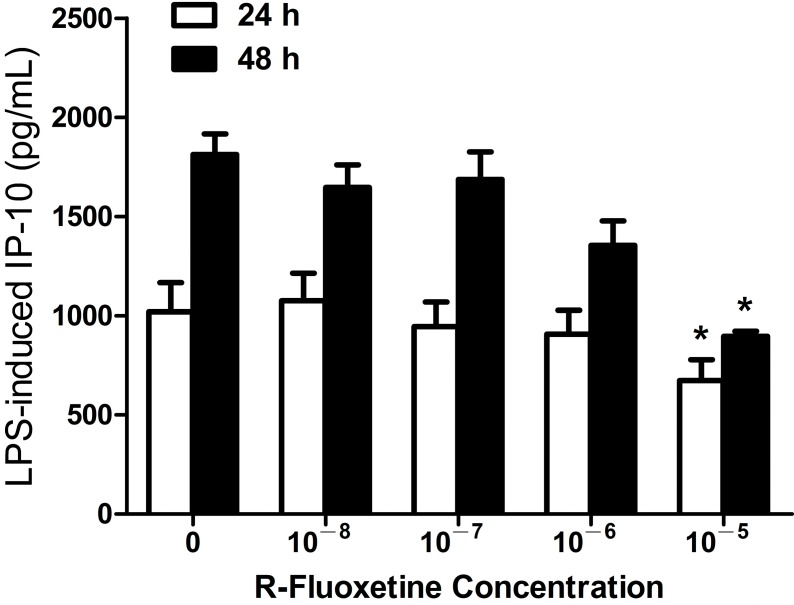
R-fluoxetine (10^−5^ M) suppressed LPS-induced IP-10 expression in THP-1 cells at 24 and 48 h after LPS (0.2 µg/mL) stimulation. * *p* < 0.05 compared with the control (LPS-untreated cells).

We next investigated whether R-fluoxetine would have a suppressive effect on IP-10 expression in monocytes, similar to S-fluoxetine. We found that R-fluoxetine did suppress LPS-induced IP-10 production in THP-1 cells (10^−5^ M after 24 and 48 h of LPS stimulation, both *p* < 0.05) (Figure 2). R-fluoxetine alone had no effect on IP-10 production (data not shown).

#### 2.1.3. Bupropion Suppressed LPS-Induced IP-10 Expression in THP-1 Cells

**Figure 3:**
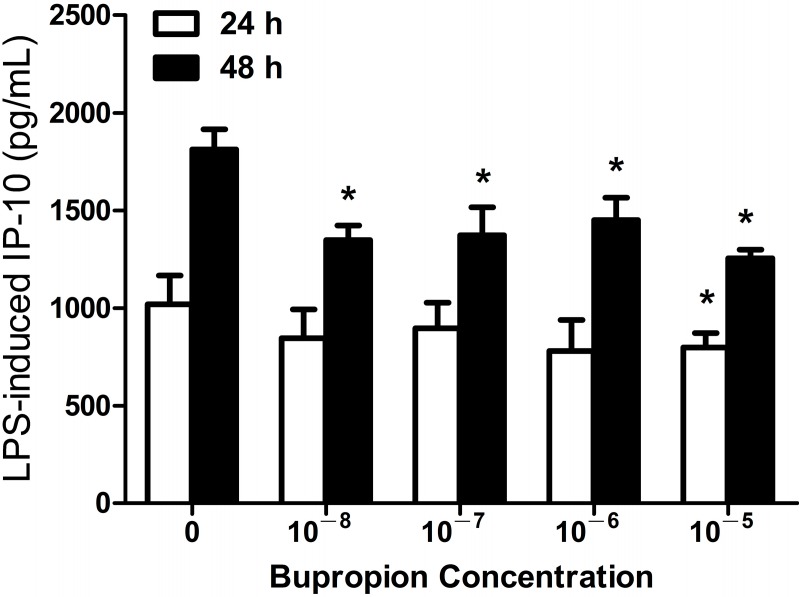
Bupropion suppressed LPS-induced IP-10 expression in THP-1 cells at 24 h (10^−5^ M) and 48 h (10^−8^–10^−5^ M) after LPS (0.2 µg/mL) stimulation. * *p* < 0.05 compared with the control (LPS-untreated cells).

Next, we examined the effect of another antidepressant, bupropion, on the expression of IP-10 in human monocytic cell lines. We found that LPS-induced IP-10 production in THP-1 cells was significantly suppressed, in a dose-dependent manner, in the presence of bupropion (10^−8^–10^−5^ M after 48 h of LPS stimulation, all *p* < 0.05) (Figure 3). Bupropion alone had no effect on IP-10 production (data not shown).

#### 2.1.4. Imipramine, Moclobemide, Venlafaxine and Mirtazapine Had no Effect on LPS-Induced IP-10 Expression in THP-1 Cells

Since S-fluoxetine, R-fluoxetine and bupropion could significantly suppress LPS-induced IP-10 expression in human monocytes, we next examined whether other commonly-used antidepressants had similar effects. We found that imipramine, moclobemide, venlafaxine and mirtazapine had no effect on LPS-induced IP-10 expression in THP-1 cells (data not shown).

#### 2.1.5. Fluoxetine and Bupropion Had no Cytotoxic Effect on THP-1 Cells

**Figure 4:**
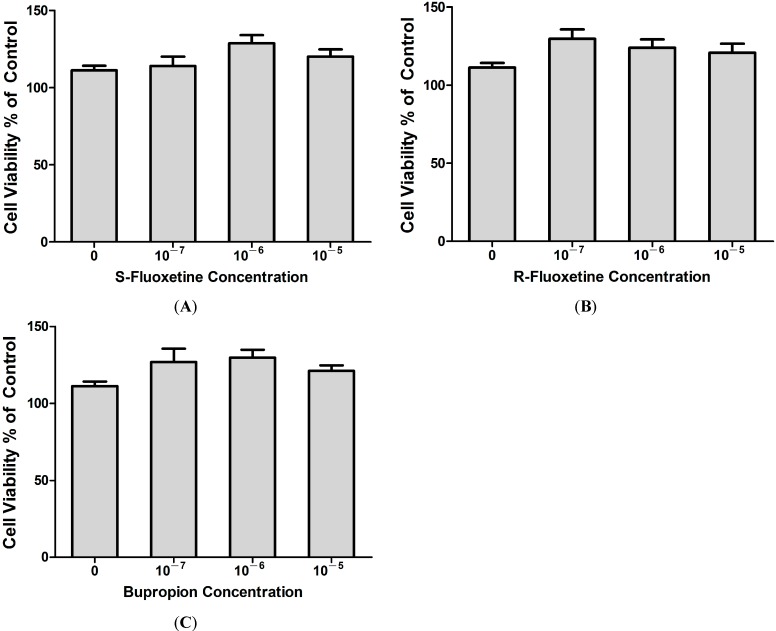
S-fluoxetine (0–10^−5^ M) (**A**); R-fluoxetine (0–10^−5^ M) (**B**) and bupropion (0–10^−5^ M) (**C**) had no cytotoxic effects in THP-1 cells led to suppressive effects of IP-10 expression after LPS (0.2 µg/mL) stimulation.

We next investigated whether the suppressive effect of S-fluoxetine, R-fluoxetine and bupropion on LPS-induced IP-10 expression resulted from a cytotoxic effect on THP-1 cells. The XTT cell proliferation assay was used to determine the effect of the three antidepressants on THP-1 cell proliferation. Results showed that S-fluoxetine (Figure 4A), R-fluoxetine (Figure 4B) and bupropion (Figure 4C) were not cytotoxic to THP-1 cells.

#### 2.1.6. S- and R-Fluoxetine Suppressed LPS-Induced IP-10 via the Mitogen-Activated Protein Kinase (MAPK)-p38 Pathway

**Figure 5:**
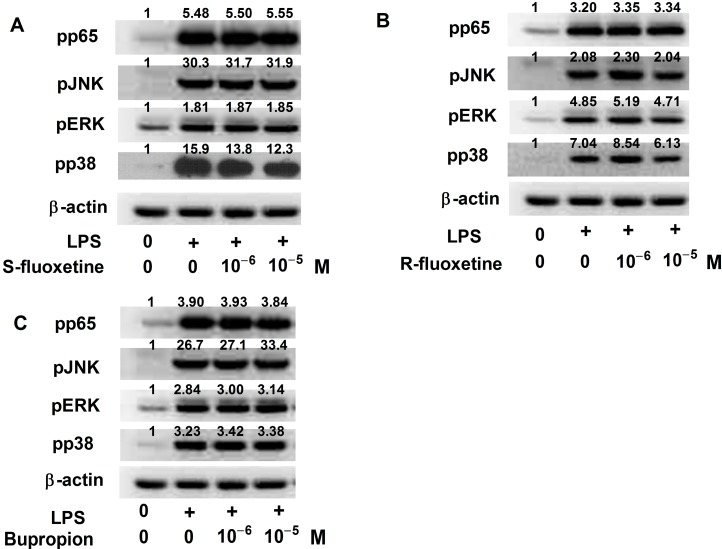
S-fluoxetine (**A**) and R-fluoxetine (**B**); but not bupropion (**C**), suppressed LPS-induced pp38 expression. S-fluoxetine, R-fluoxetine and bupropion had no effect on LPS-induced pp65, pJNK and pERK expression. Relative optic density of each band on Western blot to the control group was labeled.

We previously reported that the expression of IP-10 induced by LPS in THP-1 cells involved the MAPK and NF-κB pathways [25]. We next examined whether the suppressive effect of S-fluoxetine, R-fluoxetine and bupropion on LPS-induced IP-10 expression occurred through the MAPK or NF-κB-p65 pathway. Western blot showed that S- and R-fluoxetine suppressed LPS-induced phosphorylation of p38, but not JNK, ERK or NF-κB-p65 expression (Figure 5A,B). However, bupropion had no effect on LPS-induced phospho-p65 and phospho-JNK, and phospho-ERK and phospho-p38 expression (Figure 5C).

### 2.2. Discussion

To the best of our knowledge, the present study is the first to provide evidence that six different classes of antidepressants influence IP-10 chemokine expression in LPS-stimulated monocytes, and also to elucidate the detailed intracellular mechanism. Our data show that fluoxetine and bupropion inhibited the inflammatory process of human primary monocytes. They both significantly reduced the IP-10 chemokine production on LPS-stimulated THP-1 cells in human primary monocytes. Furthermore, the suppressive effect of fluoxetine on LPS-induced IP-10 expression was through the MAPK-p38 pathway. These findings are worthy of further discussion.

Modern diseases such as depression, CVD, stroke, diabetes, and dementia are all characterized by reliable inflammatory activity. A minor increase in inflammation—such as that observed in depression—is enough to strongly predict the development over time of many of these modern disease states [26,27,28,29]. As depression and CVD are both related to inflammation, patients with both conditions may represent a subset with the most extensive disease. Hence, it is conceivable that depression in combination with CVD increases the risk of an adverse outcome [6]. Knowledge of potential treatments and related mechanisms is urgently needed. Only certain antidepressants may reduce the mortality of CVD patients with comorbid depression [6,30,31], as discussed below.

Several recent large-scale studies have reported that SSRI treatment for depression may effectively decrease cardiovascular mortality in depressed patients with CVD [32,33]. The Enhancing Recovery in Coronary Heart Disease (ENRICHD) clinical trial [33] demonstrated that SSRI use was associated with a 43% reduction in the risk of death or nonfatal myocardial infarction (MI), and a 43% lower risk of all-cause mortality during a follow-up 29-month period. Antidepressant use by SSRI drug-type was 49.5% for sertraline, 28.9% for paroxetine, 13% for fluoxetine and 7.6% for citalopram. Another major longitudinal follow-up study of antidepressant treatment in patients with CVD, the Sertraline Antidepressant Heart Attack Randomized Trial (SADHART) [32], showed that the incidence of cardiovascular adverse events, congestive heart failure, angina or stroke was numerically greater in the placebo group (22.4%) than in the group treated with sertraline (14.5%). However, the difference failed to achieve statistical significance. In addition, a population-based case-control analysis of the UK General Practice Research Database (GPRD) was performed to evaluate the association of exposure to different groups of antidepressants with the risk of AMI [34]. The SSRIs they investigated were citalopram, fluoxetine, fluvoxamine, paroxetine, sertraline and venlafaxine. The analysis provides further evidence that the use of SSRIs is associated with a slightly decreased risk of AMI. Adjusted ORs for the current use of SSRIs, non-SSRIs, or other antidepressants, compared with non-use of antidepressants, were 0.63, 0.92 and 0.59, respectively.

The mechanisms by which SSRI treatment led to reduced cardiovascular mortality remain unclear. Authors that had previously performed analyses of the SADHART and ENRICHD trials proposed that the reduced mortality was related to the fact that SSRIs might attenuate platelet activation by serotonin depletion in the platelets. When serotonin platelet abnormalities in depressed patients were effectively treated by SSRIs, the atherogenic risk lessened. We proposed another possible mechanism, in which antidepressants with protective effects against CVD may modulate effects on Th1-related chemokine IP-10 expression in human monocytes, because IP-10 is a potent endogenous inhibitor of angiogenesis and greatly contributes to manifestations of atherosclerosis during cardiovascular inflammations [22,24]. Clinical studies have also indicated that the elevated serum IP-10 level of CVD patients was consistently associated with the risk of CVD, such as cardiac dysfunction [35,36,37]. Our data revealed that bupropion, in a dose-dependent manner, suppressed LPS-induced IP-10 expression in THP-1 cells (Figure 3), but only high-dose fluoxetine suppressed this expression (Figure 1 and Figure 2). The result implies that buproprion, *in vitro*, might be more effective than fluoxetine as a cardioprotective agent. Therefore, clinical evidence supports the cardioprotective effects of fluoxetine and buproprion [30,32,33,34]. Determining the clinical efficacy of fluoxetine and buproprion in cardiovascular protection is worthy of further clinical investigation.

Atherosclerosis, the background for many CVDs, is now thought to be a chronic inflammatory response to arterial injury that is characterized by accumulation of lipid and fibrotic entities due to hypercholesterolemia or the shear stresses of hypertension or disordered blood flow [38,39,40,41]. The LPS stimulation of monocytes activates complex signaling pathways that are important links between CVD development and the immune system. The most ubiquitous and best understood pathways, including the IKK-NF-κB pathway and the MAPK pathways, in turn activate a variety of transcription factors, which coordinate the induction of many gene encoding inflammatory mediators [21]. Moreover, LPS stimulates the activation of various MAPK pathways, including the p38, JNK and ERK pathways; these pathways subsequently directly or indirectly phosphorylate and activate various transcription factors, such as Elk-1, c-Jun, c-Fos, ATF-1 or ATF-2 [21]. Thus, our study demonstrated that the suppressive effect of S- and R-fluoxetine on LPS-induced IP-10 expression was via the MAPK-p38 pathway, without the involvement of the MAPK-JNK and -ERK pathways. Evidence has been accumulating from preclinical investigations and clinical trials that activated p38 is present in a wide spectrum of cardiovascular pathologies, and p38 inhibitors are available for treatment of CVD [42,43,44]. Hence, the complex pathways contributing to the suppressive effect of bupropion on LPS-induced IP-10 expression remain unclear.

Limitations

Although this study demonstrated the critical influence of Th1-related chemokine IP-10 expression in the human monocytic cell line, THP-1 (American Type Culture Collection, Rockville, MD, USA), there may be differences between the blood of normal controls and that of CVD patients with depression. Future investigation will be required to solidify our finding in the monocytic cell line with health human or CVD patients.

## 3. Experimental Section

### 3.1. Reagents and Cell Preparation

Six different classes of antidepressants, including R- and S-fluoxetine (SSRIs), imipramine (TCA), moclobemide (RIMA), venlafaxine (SNRI), bupropion (norepinephrine-dopamine reuptake inhibitor, NDRI), and mirtazapine were purchased from Sigma-Aldrich company (Sigma-Aldrich, St. Louis, MO, USA). These antidepressants were initially dissolved in absolute water (hydrogen oxide) or dimethyl sulfoxide with medium and then freshly made from original stock from a minimum concentration of 10^−8^ M to a maximum concentration of 10^−5^ M [45].

The human monocytic cell line, THP-1 cells (American Type Culture Collection, Rockville, MD, USA), was cultured in RPMI 1640 medium (Sigma-Aldrich, St. Louis, MO, USA) supplemented with 10% fetal bovine serum (Sigma-Aldrich, St. Louis, MO, USA), 100 U/mL of penicillin (Sigma-Aldrich, St. Louis, MO, USA), and 100 µg/mL of streptomycin (Sigma-Aldrich, St. Louis, MO, USA) at 37 °C with 5% CO_2_ in a humidified incubator. THP-1 cells were centrifuged and resuspended in fresh media in 24-well round-bottom plates at a concentration of 2 × 10^5^/mL for 24 h before experimental use. The cells were pretreated with R-fluoxetine (10^−8^–10^−5^ M), S-fluoxetine (10^−8^–10^−5^ M), imipramine (10^−8^–10^−5^ M), moclobemide (10^−8^–10^−5^ M), venlafaxine (10^−8^–10^−5^ M), bupropion (10^−8^–10^−5^ M), or mirtazapine (10^−8^–10^−5^ M), 2 h before LPS (0.2 µg/mL; *Escherichia coli* derived; Sigma-Aldrich, St. Louis, MO, USA) stimulation. Cell supernatants were collected 24 and 48 h after LPS stimulation.

### 3.2. Cell Viability Tests

THP-1 cells were incubated with various concentrations of R-fluoxetine, S-fluoxetine and bupropion in 96-well plates for 24–48 h. The XTT cell proliferation assay (Biological Industries Ltd., Kibbutz Beit Haemek, Israel) was used to determine cytotoxicity, following the manufacturer’s instructions. Cell viability was expressed as a percentage of the control group.

### 3.3. Western Blotting Analysis

After treatment for 2 h with or without R-fluoxetine (10^−6^–10^−5^ M), S-fluoxetine (10^−6^–10^−5^ M) or bupropion (10^−6^–10^−5^ M), the cells were stimulated with LPS (0.2 µg/mL) and were lysed with equal volumes of ice-cold 150-µL lysis buffer 1 hour later. After centrifugation at 13,000× *g* for 15 min, equal amounts of cell lysates from each experimental condition were analyzed by Western blot with anti-MAPK (p38, ERK and JNK) and anti-phospho-MAPK (phospho-p38, phospho-ERK and phospho-JNK) antibodies, and anti-p65 and anti-phospho-p65 antibodies (Santa Cruz Biotechnology, Santa Cruz, CA, USA). Immunoreactive bands were visualized using horseradish peroxidase-conjugated secondary antibody (Santa Cruz Biotechnology, Santa Cruz, CA, USA) and the enhanced chemiluminescence system (Amersham Pharmacia Biotech, Sunnyvale, CA, USA).

### 3.4. Enzyme-Linked Immunosorbent Assay (ELISA)

The IP-10 concentration of the cell supernatants was determined using commercially available ELISA-based assay systems (R&D system, Minneapolis, MN, USA), following the protocols recommended by the manufacturer.

### 3.5. Statistical Analysis

Experiments were performed at least in triplicate. The data are expressed as means ± standard deviation. We analyzed differences between the control and experimental groups using Student’s *t-*test. A *p* < 0.05 was considered to be statistically significant.

## 4. Conclusions

Fluoxetine and bupropion could not only treat depression but also reduce Th1-related chemokine IP-10 production in monocytes. Our results may indicate a possible mechanism regarding how particular antidepressants reduce the risk of cardiovascular disease (CVD). The novel finding in the present study is worthy of further clinical research to examine the relevance of *in vivo* effects. The new-generation SSRIs not only are considered to be free from the cardiotoxicity of their predecessors [12], but can also be considered as safe and efficacious agents against depression, platelet activation, atherosclerosis and the development and prognosis of coronary heart disease [13]. The nature and extent of the relationship between depression and CVD has yet to be fully established. Even though we proposed a chemokine-related pathway to explain the atherosclerosis protective effect as other than platelet activation, there is a need for more clinical studies in order to establish the exact biochemical mechanisms that are responsible for these diseases and the immunoregulatory effects of chronic use of SSRI medications.
